# *Of Mice and Men:* Advances in the Understanding of Neuromuscular Aspects of Myotonic Dystrophy

**DOI:** 10.3389/fneur.2018.00519

**Published:** 2018-07-10

**Authors:** Sandra O. Braz, Julien Acquaire, Geneviève Gourdon, Mário Gomes-Pereira

**Affiliations:** ^1^Laboratory CTGDM, INSERM UMR1163, Paris, France; ^2^Institut Imagine, Université Paris Descartes—Sorbonne Paris Cité, Paris, France

**Keywords:** myotonic dystrophy, mouse, trinucleotide DNA repeat, skeletal muscle, cardiac muscle, central nervous system, brain, RNA toxicity

## Abstract

Intensive effort has been directed toward the modeling of myotonic dystrophy (DM) in mice, in order to reproduce human disease and to provide useful tools to investigate molecular and cellular pathogenesis and test efficient therapies. Mouse models have contributed to dissect the multifaceted impact of the DM mutation in various tissues, cell types and in a pleiotropy of pathways, through the expression of toxic RNA transcripts. Changes in alternative splicing, transcription, translation, intracellular RNA localization, polyadenylation, miRNA metabolism and phosphorylation of disease intermediates have been described in different tissues. Some of these events have been directly associated with specific disease symptoms in the skeletal muscle and heart of mice, offering the molecular explanation for individual disease phenotypes. In the central nervous system (CNS), however, the situation is more complex. We still do not know how the molecular abnormalities described translate into CNS dysfunction, nor do we know if the correction of individual molecular events will provide significant therapeutic benefits. The variability in model design and phenotypes described so far requires a thorough and critical analysis. In this review we discuss the recent contributions of mouse models to the understanding of neuromuscular aspects of disease, therapy development, and we provide a reflective assessment of our current limitations and pressing questions that remain unanswered.

## Introduction

Animal models offer experimental tools to investigate the causes and mechanisms of disease, when the access to human samples is limited. The remarkable progresses in genetic engineering allowed the introduction of human mutations in the mouse genome, to reproduce molecular, cellular and physiological disease manifestations. The resulting phenotypes provide insight to confirm starting hypotheses, reveal novel pathogenic mechanisms and evaluate new therapies. Myotonic dystrophy (DM) illustrates the cardinal contribution of mouse models to the systematic dissection of a complex disease mechanism, from genetic mutation to the design of clinical trials.

DM is the most common form of adult muscular dystrophy, characterized by pleiotropic symptoms, which are highly variable in their nature and severity (1). Major muscular features include myotonia, muscle weakness, atrophy and smooth muscle dysfunction. Cardiac conduction defects and arrhythmias are associated with cardiomyopathy and may lead to sudden death (1). Brain involvement is illustrated by predominant structural abnormalities of the white matter, cognitive impairment (such as executive dysfunction, visuospatial deficits and abnormal social cognition), behavioral changes (such as apathy and social avoidance) and excessive daytime sleepiness (2). Other peripheral disease manifestations include insulin resistance, iridescent posterior subcapsular cataracts, and gastrointestinal complications (such as constipation/diarrhea) (1).

Two different autosomal dominant mutations in two unrelated genes cause DM and define two genetically distinct forms of the condition. DM type 1 (DM1) is caused by the expansion of a CTG trinucleotide repeat in the 3′-untranslated region (UTR) of the DM protein kinase (*DMPK*) gene (3). The DM type 2 (DM2) mutation consists in the expansion of an intronic CCTG tetranucleotide in the CCHC-type zinc finger nucleic acid binding protein (*CNBP*) gene (4). Although genetically distinct, DM1 and DM2 share a toxic RNA gain of function mechanism. In both conditions, expanded CUG/CCUG transcripts accumulate in the cell nucleus to form RNA aggregates or RNA *foci* (4–6), which perturb the function of RNA-binding proteins and a number of downstream events (7). Although clinically similar, disease symptoms are usually milder in DM2 than in DM1 (1, 8).

In a scenario where the expansion of simple non-coding DNA repeats has a broad deleterious impact on multiple tissues and physiological processes the generation of mouse models that faithfully reproduce the disease presents unique challenges. In order to be clinically relevant mouse models must have *construct, face* and *predictive* value (9). In other words, relevant mouse models must recapitulate the genetics and molecular pathogenesis (construct value); they must mimic clinical human features, both molecularly and physiologically (face value); and they must provide a platform to determine the effectiveness of new therapeutic interventions on a clinical population (predictive value). However, mouse models rarely, if ever, completely recapitulate all aspects of human disease. This is particularly applicable to DM, given the clinical variability of the disease, the involvement of multiple tissues and the complexity of the underlying molecular pathways. Even with this caveat, mouse models, alone or in combination, have been instrumental to understand fundamental molecular pathomechanisms (10). Importantly, they have allowed molecular and cellular analyses at various developmental stages, as well as in cell types and tissues that are not easily accessible in humans. We have previously reviewed the contribution of mouse models to decipher the grounds of RNA toxicity and to evaluate promising preclinical assays (10), but there is little doubt that mouse models have continued to provide in-depth understanding of DM disease mechanisms over the last years.

Here we discuss how recent mouse data refined our understanding of RNA toxicity and unfolded numerous roles and pathogenic implications of the RNA-binding proteins dysregulated in DM. We review other emerging disease intermediates and dysregulated signaling pathways recently uncovered. Pre-clinical therapeutic developments are discussed in light of their contribution to reinforce fundamental aspects of disease pathogenesis. We focus primarily on the neuromuscular aspects of the disease to establish correlations between mouse data and human pathology. We point out some contradictory findings between mouse models to illustrate the challenges, complexity and variability of DM disease pathogenesis.

## From DNA repeats to toxic RNA transcripts

The toxicity of RNA repeats was unequivocally demonstrated in HSA^LR^ transgenic mice, through the insertion of an expanded CTG sequence in the 3′UTR of an unrelated gene: the human actin, alpha 1 (*ACTA1*) gene. The expression of CUG-containing *ACTA1* transcripts in mouse skeletal muscle generated genuine myotonia and histological signs of myopathy (11). The elimination of the expanded transcripts by antisense oligonucleotides reduced myotonia in these mice (12), confirming the toxicity of CUG RNA repeats.

The absence of muscle weakness in the HSA^LR^ mouse line that expressed the highest transgene levels and showed pronounced muscle histopathology was intriguing and suggested the dissociation between the toxicity of RNA foci and the etiology of muscle weakness (11), an hypothesis that persisted for some years. However, the later analysis of a second HSA^LR^ line, which also expressed high levels of the transgene and showed myotonia, revealed reduced grip strength (13). Contrary to the initial reports, these findings corroborate the view that the expression of toxic RNA repeats is sufficient to trigger muscle weakness. CUG RNA toxicity was further demonstrated and confirmed in other mouse lines, listed in Table 1.

**Table 1:** Summary of transgene design and expression in the DM mouse models most extensively studied.

**Models of toxic RNA expression (poly-CUG models)**					
**Mouse model**	**(CTG)n**	**Flanking sequence**	**Promoter**	**Tissue expression**	**References**
HSA^LR^	~250	Human skeletal actin 3′UTR	Human *ACTA1*	Skeletal muscle	(11)
DMSXL	>1,000	Human DMPK locus	Human *DMPK*	Ubiquitous	(14, 15)
EpA960	960	Human DMPK 3′UTR	CMV	Inducible (ubiquitous or tissue-specific)	(16)
DM5/DM200	5/200	Tet-responsive, human *DMPK* promoter	Human *DMPK*	Inducible (ubiquitous or tissue specific)	(17)
**Models of altered RNA-binding proteins**					
**Mouse model**	**Mutation/construct**		**Tissue expression**		**References**
*Mbnl1* KO	Constitutive deletion of *Mbnl1* exon 3		Ubiquitous		(18)
*Mbnl2* KO	Constitutive deletion of *Mbnl2* exon 3		Ubiquitous		(19)
*Mbnl3* KO	Constitutive deletion of *Mbnl3* exon 3		Ubiquitous		(20)
*Mbnl1/Mbnl2* DKO	Constitutive deletion of *Mbnl1* exon 3 Constitutive or conditional deletion of *Mbnl2*		Ubiquitous deletion of *Mbnl1*. Ubiquitous or tissue-specific deletion of *Mbnl2*		(21)
*Mbnl1/Mbnl3* DKO	Constitutive deletion of *Mbnl1* exon 3 Constitutive deletion of *Mbnl1* exon 2				(22)
*Mbnl1/Mbnl2/Mbnl3* TKO	Constitutive deletion of *Mbnl1* exon 3 Conditional deletion of *Mbnl2* and *Mbnl3*		Ubiquitous deletion of *Mbnl1*. Tissue-specific deletion of *Mbnl2* and *Mbnl2*		(23)
TRECUGBP1	Human CELF1 sequence downstream of Tet-responsive CMV promoter		Inducible (ubiquitous or tissue-specific)		(24)
TRECUGBP2	Human CELF2 sequence downstream of Tet-responsive CMV promoter		Inducible (ubiquitous or tissue-specific)		(25)

The ubiquitous expression of expanded *DMPK* transcripts from the human DM1 locus resulted in multisystemic phenotypes in DMSXL mice carrying more than 1000 CTG repeats. These phenotypes include reduced muscle strength, lower motor performances, peripheral neuropathy, respiratory impairment, abnormal cognition and behavior, and cardiac conduction defects (26–29). Similarly, the inducible expression of a large, interrupted CTG repeat flanked by the 3′UTR of the *DMPK* gene produced cardiac, muscular and neurological phenotypes in EpA960 mice (16, 30, 31). Surprisingly, high expression of short (CTG)_5_ repeats within the *DMPK* 3′UTR was pathogenic in DM5 mice, causing DM1-like myotonia and cardiac conduction defects (17). Hence, the expression of many copies of a short CUG repeat may have functional outcomes that are comparable to the expression of a few copies of large CUG RNA repeats. In other words, the toxicity of repetitive RNA is two-fold: it is determined not only by the sequence length but also by the abundance of the repeat transcripts in the cell. While HSA^LR^, DMSXL and EpA960 animals accumulate foci, nuclear RNA aggregates were not detected in DM5 mice, raising the possibility that submicroscopic RNA foci can cause disease, or that soluble CUG RNA is also pathogenic (32). The DM1 molecular hallmarks reported in the main poly-CUG mouse models are summarized in Table 2. No poly-CCUG DM2 mouse model has been fully characterized yet.

**Table 2:** Molecular hallmarks of RNA toxicity in the mouse models expressing CUG RNA repeats.

**Models of toxic RNA expression (poly-CUG models)**					
**Mouse model**	**RNA foci**	**MBNL co-localization**	**CELF1 upregulation**	**Missplicing**	**References**
HSA^LR^	Skeletal muscle	MBNL1	Skeletal muscle	Severe in skeletal muscle	(11, 13, 33)
DMSXL	Multiple tissues	MBNL1 MBNL2	Brain (and CELF2) Trend in heart	Mild, age-dependent in multiple tissues	(26, 27)
EpA960	Skeletal muscle; Heart; CNS	MBNL1 MBNL2	Skeletal muscle Heart Brain	Severe in skeletal muscle and heart. Mild in brain	(16, 30, 31)
DM5/DM200	Absent	Not detected	Skeletal muscle. Normal levels in heart	Mild in skeletal muscle. Absent in heart	(17)

**Figure 1:**
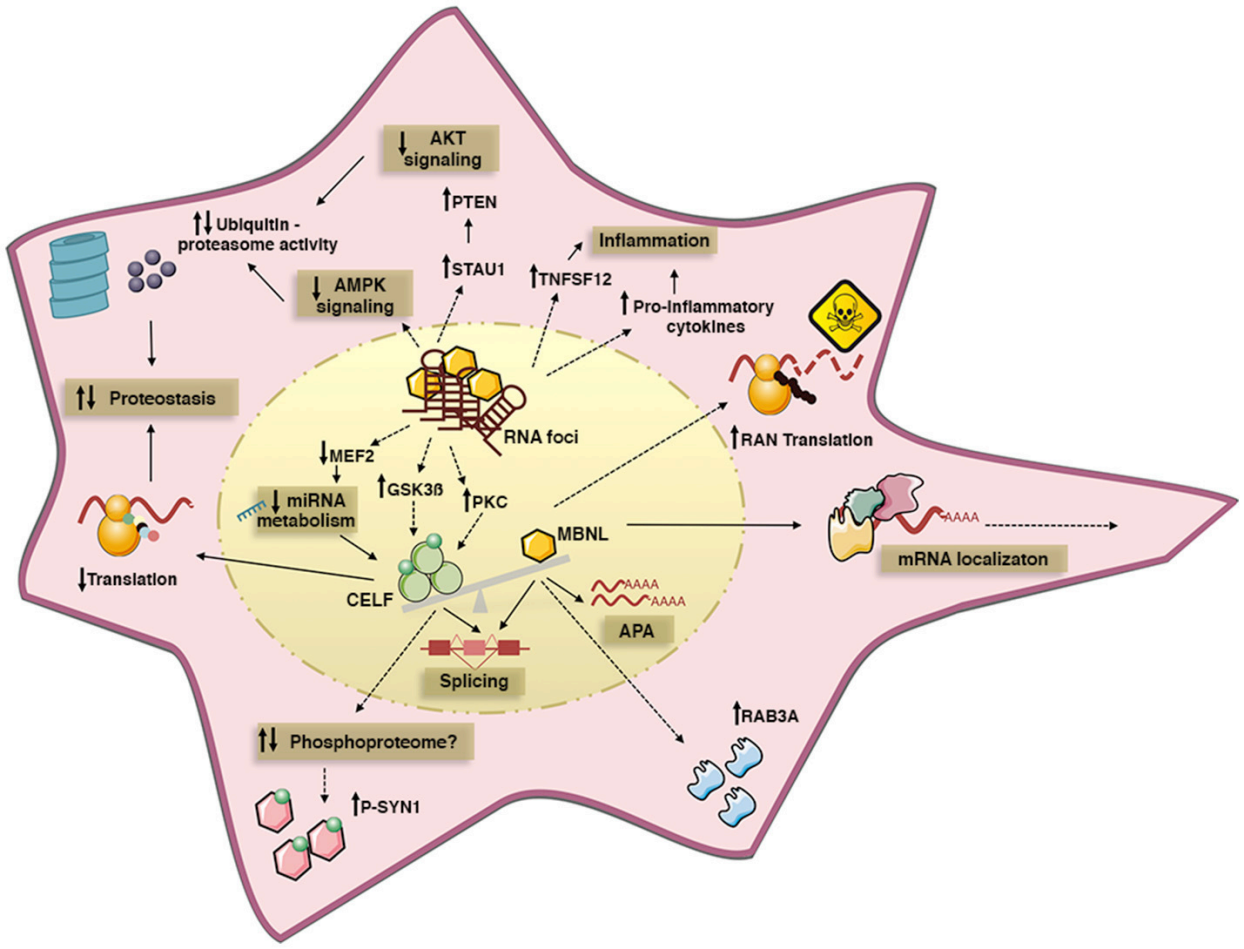
Summary of some of the cell pathways and signaling cascades dysregulated by toxic RNA repeats in DM cells. The expression of toxic RNA transcripts sequesters MBNL proteins into nuclear RNA foci, and upregulates CELF proteins. Different mechanisms may account for CELF upregulation, such as altered PKC and GSK3ß kinase activity, or changes in miRNA levels due to altered MEF2 transcription program. MBNL inactivation and CELF gain-of-function cause pathogenic missplicing. Functional MBNL inactivation alone disrupts alternative polyadenylation and intracellular localization of mRNA targets; it is also believed to dysregulate protein expression, independently of splicing, and to promote RAN translation of toxic peptides. In turn, CELF1 upregulation affects translation efficacy and it may affect the phosphorylation of a subset of proteins through unidentified mechanisms. Protein homeostasis is also perturbed by the downregulation of AKT and AMPK signaling pathways, which likely promotes protein catabolism by increased ubiquitin-proteasome activity, hence contributing to muscle atrophy and weakness. Finally, the increased expression of pro-inflammatory cytokines suggests ongoing inflammation in DM. Solid lines represent well defined disease mechanisms, while dashed lines represent circumstantial data with poorly defined mechanistic links.

RNA foci are dynamic ribonucleoproteic structures that disrupt important RNA-binding proteins (Figure 1). Members of the MBNL (muscleblind-like) family of splicing factors are sequestered and partially inactivated by the RNA foci in DM1 and DM2 (34, 35), while CELF (CUGBP Elav-like family) proteins are abnormally upregulated, at least in DM1 (16, 36, 37). MBNL sequestration, CELF upregulation and missplicing have been detected to different extents in mouse models expressing poly-CUG RNA transcripts (Table 2). MBNL and CELF proteins bind independently to RNA targets and functionally compete to regulate their downstream processing (25). The two protein families comprise key regulators of developmental splicing transitions. The combined MBNL sequestration and CELF upregulation results in the pathogenic expression of fetal isoforms in adult DM tissues (7). In other words, DM spliceopathy does not produce “unusual” splicing isoforms; instead, it is associated with the expression of normal splicing products that are not well-suited to adult tissue function, leading to the onset of typical disease manifestations. In this context myotonia is the consequence of the abnormal splicing of the CLCN1 chloride channel (38, 39), while insulin resistance is most likely associated with the missplicing of the insulin receptor (38, 40). It is important to note the significant overlap between the splicing abnormalities in DM and other muscular dystrophies (41). The similarities depict a scenario in which splicing dysregulation in DM is not only a primary disease process, but also a secondary event caused by general tissue degeneration.

In addition to the canonical sense transcripts, both DM1 and DM2 loci produce antisense transcripts, a feature shared with many microsatellite repeat loci and that has been suggested to regulate local gene expression (42). The CTG expansion interferes with the relative levels of sense and antisense RNA in DM1 patients (43) and in transgenic mice carrying the human DM1 locus (44). The pathogenic impact of these changes on local gene expression and disease mechanisms requires further studies in experimental models.

## The multifaceted role of MBNL proteins in DM pathogenesis

Humans and mice (as well as most vertebrates) express three *MBNL* genes (*MBNL1, MBNL2*, and *MBNL3*) (45). Endogenous MBNL1 and MBNL2 co-localize with CUG and CCUG RNA foci in DM1 and DM2 cells, respectively (4, 46–48), whereas MBNL3 protein was not detected in adult tissues (48).

The three *MBNL* paralogs show differences in spatial distribution in adult mouse tissues. *Mbnl1* and *Mbnl2* transcripts are ubiquitously expressed, but *Mbnl1* RNA levels are higher in heart, whereas *Mbnl2* is more homogenously distributed (49). The steady-state levels of MBNL2 protein, however, are low in adult skeletal muscle (19). *Mbnl3* transcript levels are very low in adult mice (49). Differences in protein distribution extend to cell types: the analysis of primary mouse cultures revealed higher relative levels of MBNL1 in astrocytes, while MBNL2 was more abundant in primary neurons (50).

The involvement of MBNL proteins in DM was tested in knockout lines generated either through the deletion of *Mbnl* genes alone, or the combined inactivation of multiple *Mbnl* genes (Table 1). These mice revealed some degree of functional specialization between individual members of the MBNL family and clarified their roles in disease molecular pathogenesis.

### Functional specialization of MBNL proteins: insight from single knockout lines

Direct evidence of detrimental MBNL sequestration was provided by the generation of *Mbnl1* KO mice. *Mbnl1* inactivation impacted primarily the skeletal muscle and caused pronounced myotonia, but it also resulted in DM1-like subcapsular cataracts, lack of motivation and apathy in knockout mice (18, 51). The impact on cardiac function was less obvious and dependent on the genetic background of *Mbnl1* KO mice: cardiac conduction defects were more pronounced on a homogenous 129/Sv background (52), relative to a mixed 129/Sv x C57BL6 background (21). The reasons behind strain-specific cardiac differences between the homogenous and the mixed background have not yet been resolved, but the comparison between these two lines may provide unique insight into the modifiers of disease severity. It is important to note that DM is a highly variable condition, and that variability in disease manifestations may be explained by a complex interplay between genetic modifiers and environmental factors. The backcrossing of different mouse models onto different genetic backgrounds may facilitate the identification of relevant genetic modifiers of disease.

Although reproducing critical muscular and cardiac phenotypes, *Mbnl1* KO mice did not develop prominent muscle weakness/wasting or marked cognitive deficits, aside from decreased motivation (51). Additional MBNL members may therefore serve as key disease intermediates. Indeed, the inactivation of *Mbnl2* yielded mild muscle pathology, but marked CNS phenotypes, suggesting a tissue-specific impact of *Mbnl* gene inactivation. Neurological phenotypes of *Mbnl2* KO include sleep disturbance, defective spatial memory, abnormal synaptic plasticity and seizure susceptibility (19).

**Figure 2:**
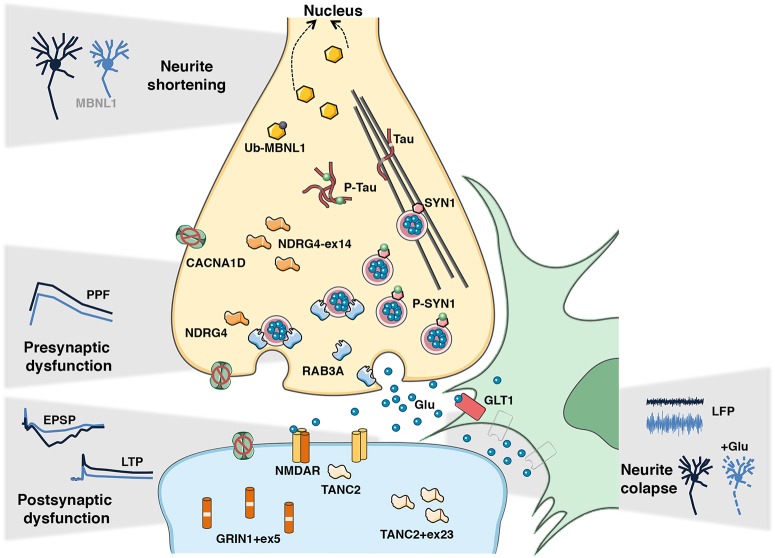
Candidate disease intermediates of DM synaptic dysfunction and learning deficits. The dysregulated disease mechanisms in the CNS of DM1 mouse models appear to involve both pre- and postsynaptic events, which lead to global synaptic dysfunction and consequent cognitive and memory deficits. In the pre-synaptic compartment the hyperphosphorylation of SYN1 and upregulation of RAB3A, together with the missplicing of *Mapt/Tau, Ndrg4*, and *Cacna1d* may contribute to impaired short-term synaptic plasticity, notably through decreased paired-pulse facilitation (PPF) detected in DMSXL mice. In the postsynaptic counterpart, the missplicing of *Grin1, Tanc2*, and *Cacna1d* may disrupt the functioning of the voltage-gated NMDA receptor, and consequently NMDAR-mediated mechanisms of long-term potentiation (LTP) detected in *Mbnl2* KO and EpA960 mice. Reduced GLT1 levels in neighboring astrocytes likely result in neuronal hyperexcitability, demonstrated by increased local field potentiation (LFP) in DMSXL mice, and it can ultimately lead to neuronal damage and neurite collapse in the presence of excessive glutamate. The mislocalization of MBNL1 into the nucleus following abnormal de-ubiquitination decreases neuritogenesis and affects neuronal morphology in EpA960 mice. Together these events likely mediate defective synaptic transmission and abnormal brain connectivity behind DM cognitive and behavioral changes.

The deleterious effect of *Mbnl1* inactivation on muscle physiology was accompanied by splicing defects that are more severe in skeletal muscle and in heart than in the CNS (18, 52, 53), and it related to the role of MBNL1 in the control of fetal-to-adult splicing transitions in muscle (18, 33). Similarly, MBNL2 appears to serve a similar function in the CNS (19). As a result of this regional specialization, *Mbnl1* KO mice express embryonic splicing isoforms predominantly in the muscle (18, 33), while *Mbnl2* KO mice exhibit embryonic splicing profiles mainly in the CNS (19). Still, we cannot exclude other significant roles of MBNL1 in the CNS independent of splicing: MBNL1 controls the steady-state levels of RAB3A, and possibly other synaptic proteins (27, 54); and it also determines the length of neuronal dendrites and axons (31) (Figure 2).

The inactivation of *Mbnl3* yielded intriguing results: despite low *Mbnl3* expression in adult muscle, *Mbnl3* KO mice exhibited reduced grip strength and age-dependent decline in skeletal muscle regeneration (20). Other age-associated phenotypes were described in an independent *Mbnl3* KO line, such as glucose intolerance, cardiac deficits and subcapsular cataracts (55). MBNL3 loss of function may therefore contribute to the accelerated aging suggested in DM (56). Interestingly, the phenotypes of *Mbnl3* KO mice are not accompanied by significant changes in alternative splicing (20, 55). Together with the primary localization of MBNL3 in the cytoplasm (23), these findings predict roles of MBNL proteins other than splicing regulation.

### Combined inactivation of MBNL proteins

Despite the significant phenotypes of *Mbnl1* and *Mbnl2* single gene knockout lines, they do not model the full disease spectrum, possibly due to compensatory mechanisms of the remaining *Mbnl* genes (21). In *Mbnl1* KO mice, *Mbnl2* expression is upregulated and MBNL2 protein binds to target transcripts that are normally regulated by MBNL1 (21). In order to recreate a situation that resembles more closely the human disease, in which the three MBNL paralogs are sequestered by toxic RNA foci (35), compound knockout mice were generated (Table 1). While *Mbnl1/Mbnl2* double knockout (DKO) mice were embryonic lethal, the inactivation of one *Mbnl2* copy in a *Mbnl1* KO background was sufficient to exacerbate myotonia and trigger muscle weakness, loss of mature neuromuscular junctions and cardiac conduction defects, which were absent in single *Mbnl1* KO mice (21, 57). The aggravated phenotypes were accompanied by an increasing severity in spliceopathy and significant changes in alternative polyadenylation (APA) (58). The molecular analysis of *Mbnl1/Mbnl2* DKO mice was instrumental to reveal the role of MBNL proteins in the regulation of APA: MBNL proteins and the APA machinery compete to bind to APA sites of a subset of transcripts, in a mechanism that regulates the processing and length of the 3′ end of target transcripts, with subsequent implications for their stability and localization (Figure 1). Similar to DM splicing abnormalities, the sequestration and functional inactivation of MBNL proteins by toxic RNA results in the persistence of fetal APA profiles in adult muscle and brain of DM1 and DM2 patients (58, 59). The direct contribution of individual APA defects to specific symptoms is unclear (60), but the mouse models available offer unique tools to address this question, not only in DM but also in other conditions in which MBNL proteins are sequestered by toxic RNA repeats.

Dual depletion of *Mbnl1* and *Mbnl3* also enhanced myotonia, muscle weakness and myopathy in skeletal muscle (22). However, in contrast to *Mbnl1/Mbnl2* DKO, increased myotonia was not associated with a greater extent of splicing dysregulation in *Mbnl1/Mbnl3* DKO mice. Instead, enhanced myotonia was the result of the synergy between *Clcn1* missplicing caused by *Mbnl1* deletion alone, and defective CLCN1 translation, caused by combined inactivation of MBNL1 and MBNL3 proteins (22).

More recently, conditional triple knockout (TKO) mice were generated by muscle-specific deletion *Mbnl2* and *Mbnl3* on an *Mbnl1* knockout background (Table 1). *Mbnl1/Mbnl2/Mbnl3* TKOs present high neonatal mortality, growth defects, respiratory distress, muscle weakness and wasting in association with pronounced splicing and gene expression defects (23). Interestingly, the total spliceopathy in muscle was only modestly increased in *Mbnl1/Mbnl2/Mbnl3* TKO mice, relative to *Mbnl1/Mbnl2* DKOs, supporting the view that congenital spliceopathy is primarily due to compound loss of MBNL1 and MBNL2, and further pointing to MBNL3 functions, other than splicing regulation. Hence, the congenital form of the disease seems to require combined inactivation of the three *Mbnl* paralogs from an early developmental stage. DMSXL mice, which also show growth retardation from birth, and DM1 individuals express both sense and anti-sense *DMPK* transcripts from embryonic and fetal stages (44), confirming that the toxic RNA mechanisms behind congenital cases could operate early on during development.

Overall the generation and characterization of single and compound *Mbnl* KO mouse models, demonstrated that the simultaneous sequestration of various MBNL proteins is instrumental for the development of clinical manifestations of DM. Given the sparse availability and technical difficulties of working with human DM tissue, constitutive and conditional *Mbnl* KO mice grant the possibility to investigate abnormal RNA processing in different tissues, cell types and developmental stages.

### Insight from MBNL replacement strategies

MBNL1 loss of function accounts more than 80% of missplicing events and nearly 70% of expression defects in the skeletal muscle of HSA^LR^ mice (61, 62), strongly anticipating the benefits of therapeutic gene replacement. Overexpression of MBNL1 through viral infection or genetic manipulation ameliorated myotonia and splicing abnormalities in the tibialis anterior of HSA^LR^ mice, however it was insufficient to fully correct muscle histopathology (63, 64). While further confirming the role of *Mbnl1* loss of function in the onset of myotonia, these findings also hint at the involvement of additional disease intermediates in muscle pathology. It is conceivable that other MBNL proteins might be necessary to fully reverse muscle phenotypes. Prior to the further development of MBNL replacement strategies, it is important to evaluate to which extent MBNL proteins are interchangeable and capable to functionally replace each other in muscle and in other tissues of DM1 mouse models expressing expanded CUG transcripts. Alternatively, the incomplete rescuing of muscle physiology in HSA^LR^ mice by MBNL1 overexpression points to the involvement of other families of disease intermediates alongside MBNL proteins.

### Cytoplasmic roles of MBNL: RNA trafficking and proteotoxicity

MBNL proteins are also present in the cytoplasm (23, 48), where they likely regulate mRNA stability (61, 62, 65), as well as the intracellular localization of mRNA transcripts through binding to the 3′UTR of their targets (66, 67). The role of MBNL proteins in mRNA trafficking might be particularly relevant in highly polarized brain cells, such as neurons. Altered MBNL activity or intracellular localization in DM1 could be detrimental for correct transport of mRNAs toward specialized cell compartments (such as axons, dendrites and synapses), which would subsequently affect local translation and ultimately cell function.

In further support of a cytoplasmic function of MBNL proteins, the expression of CUG RNA in the forebrain of EpA960 mice (Table 1) affects MBNL1 ubiquitination and distribution between the nucleus and the cytoplasm (Figure 2), prior to the shortening of neuronal dendrites and axons (31, 68). Morphological impairments occurred in the absence of missplicing, suggesting a contribution of cytoplasmic MBNL1 to disease process.

MBNL proteins have been recently proposed to act as guardians against proteotoxicity. CUG and CCUG RNA generate toxic peptides through non-conventional repeat-associated non-ATG (RAN) translation (69, 70). The combination of bidirectional transcription with RAN translation of expanded repeats produces multiple toxic species, which co-localize with markers of apoptosis, supporting a role in disease pathology (71). Interestingly, RNA accumulation seems to co-exist and exacerbate RAN translation in the same cell, in a mechanism mediated by MBNL proteins: MBNL sequestration and inactivation by nuclear RNA foci promotes RAN translation in DM1 and DM2 cell models (70, 72). RAN products have been reported in DMSXL mice (69). Future mouse studies are required to elucidate the relationship between RNA foci, MBNL protein and RAN translation, and the pathogenicity of RAN peptides in multiple tissues and cell types.

## CELF proteins: splicing, translation and DM pathogenesis

CELF1 upregulation correlates with muscle histopathology in DM1 patients and DM5 transgenic mice (Table 1) (73), pointing to a direct role of CELF1 in disease pathogenesis (Figure 1). The upregulation of CELF1 in DM2 skeletal muscle is contentious, with conflicting reports of normal and increased protein levels (33, 74–76).

To directly address the role of CELF1 gain of function, overexpressing mice were generated. Ubiquitous CELF1 upregulation resulted in severe developmental phenotypes and muscle histopathology (77), which correlated with transgene expression levels (77, 78). The high mortality of these mice limited their face and predictive value. Conditional mouse lines were more informative, since they offered the opportunity to focus on individual tissues and assess the pathogenic contribution of CELF protein overexpression alone (Table 1). Induction of CELF1 transgene expression in mouse skeletal muscle was sufficient to reproduce muscle wasting, defective motor performance and myopathy (79); while CELF1 upregulation in heart caused cardiac conduction defects, cardiomyopathy with hypertrophy and early mortality (24). As expected, muscular and cardiac phenotypes were accompanied by missplicing events in muscle and heart, respectively (24, 79). A splicing-mediated effect was further supported by the expression of a dominant-negative *Celf1* variant in HSA^LR^ mice, which partially corrected missplicing in skeletal muscle (80).

CELF proteins can also regulate the alternative splicing of transcripts involved in neuronal function (81). Therefore, the upregulation of CELF1 and CELF2 reported in human DM1 brains (27, 82) may have a substantial contribution to the etiology of neurological dysfunction. Conditional overexpressing models could help investigate the cognitive and behavior consequences of CELF1 or CELF2 upregulation, and identify subsets of transcripts that specifically respond to these two RNA-binding proteins. In support of target discrimination between CELF proteins, it was shown that the splicing of *MAPT* exon 10 responds specifically to CELF2, but not to CELF1 upregulation (82).

Although already generated, CELF2-overexpressing mice have only been used in molecular approaches to study the antagonistic role of MBNL and CELF proteins in splicing regulation (25). The phenotypic consequences of CELF2 overexpression have not been reported yet.

### Cytoplasmic functions of CELF proteins

In addition to regulating alternative splicing in the nucleus, CELF proteins have cytoplasmic roles in the regulation of mRNA stability, translation and deadenylation (83). The distribution of CELF1 between the nucleus and the cytoplasm is regulated by AKT phosphorylation (84). As a result, CELF gain of function in DM1 has intricate consequences that affect multiple cellular pathways in different cell compartments.

CELF1 activity is controlled by multiple phosphorylation events. The role of CELF1 in translation depends on the phosphorylation of Serine-302: phosphorylated CELF1 acts as an activator, while unphosphorylated CELF1 represses translation (84, 85). In DM1, the increase in the total levels of CELF1 is accompanied by the elevation of both phosphorylated and unphosphorylated forms of CELF1 at Serine-302 (40, 86, 87). This results in the reprogramming of protein translation, altered proteostasis and global cell stress, which ultimately affects cell function (75, 78, 84).

DM5 mice have corroborated the cytoplasmic functions of CELF1 in DM1 pathogenesis. The genetic inactivation of *Celf1* in DM5 mice did not mitigate missplicing, but instead corrected the expression of CELF1 translational targets in skeletal muscle (73); a tissue that shows CELF1 upregulation in DM5 mice (17). The molecular changes were sufficient to improve motor performance, grip strength and histopathology, but left myotonia unchanged (73). These results demonstrate the pathogenic relevance of CELF1-regulated translation, and point to CELF1-independent myotonia mechanisms in DM. Interestingly, in line with the absence of CELF1 upregulation in DM5 hearts (17), *Celf1* deletion did not ameliorate the cardiac function (73).

Finally, it is worth noting that CELF1 overexpression alone is associated with the hyperphosphorylation of Synapsin-1 in cell culture (27) (Figure 2), suggesting a contributing role of this RNA-binding protein in the regulation of the phosphoproteome.

### Mechanisms of CELF upregulation and therapeutic strategies

CELF1 upregulation in DM1 operates at protein level, since transcript load remains unchanged in skeletal muscle (73). In the heart of DM1 patients and induced EpA960 mice, upregulation correlates with CELF1 protein hyperphosphorylation, higher protein stability and increased PKC activity (87) (Figure 1). Consistent with a direct role of PKC in CELF1 metabolism, treatment of EpA960 mice with PKC inhibitors immediately after transgene induction avoided CELF1 upregulation, reduced mouse mortality and improved cardiac function (88). These findings provided pharmacological evidence of the involvement of PKC in CELF1 function and in DM1 cardiac phenotypes. Surprisingly, the genetic inactivation of *Pkc* did not lower CELF1 expression or correct histopathology in the skeletal muscle of DM5 mice (89).

Different reasons may account for the differing outcomes of CELF1 results obtained with EpA960 and DM5 mice. First, the inherent differences between the two mouse models: while in EpA960 interrupted large repeats are expressed under the control of a non-*DMPK* promoter (16), DM5 mice express short (CUG)5 RNA repeat in multiple tissues and cell types under the control of the human *DMPK* promoter (17). Second, it is conceivable that the molecular mechanisms of CELF1 upregulation differ between heart and skeletal muscle: CELF1 upregulation in the skeletal muscle might be independent of PKC. Third, off-target effects of kinase inhibition might have introduced confounding factors in the analysis. Indeed, the PKC inhibitor Ro 31-8220 used in EpA960 mice has since then been found to reduce RNA foci, release MBNL1 and correct MBNL1-dependent splicing events in a cell model of DM1 (90). Furthermore, Ro 31-8220 can also inhibit other kinases, including GSK3ß (91).

Experimental evidence of the role of GSK3ß in DM1 muscle pathology was obtained in HSA^LR^ mice: GSK3ß inhibition restored CELF1 protein levels and translational activity, improved muscle strength and corrected histopathological changes (13, 92). The possibility remains that different aspects of CELF1 metabolism are controlled by different phosphorylation events: phosphorylation by PKC increases protein stability (87, 92), GSK3ß controls the translational activity of CELF1 (13) and AKT regulates the nucleus-cytoplasm distribution of CELF1 (84) (Figure 1). We currently do not know the molecular link between the repeat expansion and altered kinase activity.

Kinase-independent mechanisms of CELF1 upregulation have been proposed. Under physiological conditions, CELF1 protein decreases in adult mouse tissues in response to a developmental increase in a subset of microRNA (miRNA) species. Transgene induction in EpA960 revealed that CUG RNA toxicity disrupts the MEF2 transcription network, lowers miRNA expression reversing the physiological miRNA developmental program and causing CELF1 upregulation (93). Therefore altered levels of miRNA in DM1 tissues could explain CELF1 upregulation.

To discard the possibility of a direct or indirect regulation of CELF1 by MBNL proteins, CELF1 expression was measured in *Mbnl1* KO mice, and revealed no changes (18). In contrast, induction of CELF1 over-expression in transgenic mice yielded MBNL1 upregulation, possibly mediated by tissue regeneration (79).

Despite progress in the understanding of the multifaceted metabolism of CELF1 in DM1, the jury is still out on the molecular mechanisms of upregulation in DM1 and the extent of the therapeutic benefits of CELF targeting in tissues, other than the heart. The mechanisms behind CELF2 upregulation in the CNS of DMSXL mice are less clear (27). Useful mouse models are available to address these questions (Table 1 and Table 2), through pharmacological or genetic manipulation of CELF1 and CELF2 levels, as well as the activity of candidate kinases and miRNA metabolism.

## Unraveling disease intermediates and pathways behind neuromuscular pathology

Additional layers of DM1 molecular pathogenesis, beyond the canonical involvement MBNL and CELF RNA-binding proteins, have emerged from recent mouse studies of muscle and heart phenotypes. Hereditary myotonia is usually caused by the malfunction of ion channels (94). In line with this view, compelling evidence has demonstrated the direct role of *CLCN1* chloride channel missplicing in the onset of DM1 and DM2 myotonia (39, 63). The mechanisms behind muscle weakness/wasting and cardiac dysfunction can be more diverse, and mediated by a combination of interacting intermediates. In this section we first discuss some critical splicing events, whose contribution to muscle and heart pathology has been corroborated by mouse studies. Then we review the emerging role of additional pathways, whose mechanistic link with MBNL and CELF canonical disease intermediates has not yet been elucidated and deserves further attention.

### The role of missplicing in muscle and heart disease: many roads leading to Rome

Progressive muscle weakness and wasting are among the most prominent clinical features of DM1, in association with centralized nuclei and myofiber atrophy, without overt regeneration, fibrosis or necrosis (1). Previous studies have shown associations between muscle weakness and MBNL1-dependent splicing of *BIN1* (95), *CACNA1S* (96) and *DMD* (97). The recreation of the DM1 missplicing of *Bin1, Cacna1s* or *Dmd* in wild-type mice, through RNA antisense technology, corroborated the contribution of these events to muscle weakness and myopathy (95–98). However, it is still unclear if the combined inactivation of multiple MBNL proteins is the sole responsible for muscle weakness. Elevation of CELF1 protein may certainly play a determinant role too, as suggested by the muscle phenotype of CELF1-overexpressing mice (30) and by the improved muscle strength following CELF1 downregulation in HSA^LR^ mice (13). Some CELF1-responsive splicing events may provide connecting dots in the mechanisms of muscle pathology: while *RYR1* missplicing alters excitation-contraction coupling in skeletal muscle (99), the shift of *PKM* splicing to an embryonic isoform results in less efficient energy production, likely associated with muscle weakness and wasting (98).

An expected role for splicing dysregulation has also been suggested in DM heart disease. In spite of the confirmed contribution of MBNL1/MBNL2 loss of function (21) and CELF1 upregulation toward cardiac conduction defects (24), the downstream disease intermediates remained elusive. MBNL1-dependent missplicing of *SCN5A* was found in the heart of DM1 patients and *Mbnl1/Mbnl2* DKO mice. When the DM1 splicing isoform is expressed in wild-type mice, it causes DM1-like cardiac conduction defects and arrhythmias (100). The influential role of SCN5A does not rule out the contribution of other yet unidentified splicing events that may reinforce heart spliceopathy and aggravate cardiac disease in DM.

### Cellular energy sensors, proteasome activity and muscle weakness

The RNA binding protein Staufen1 is significantly upregulated in DM1 muscle biopsies, in the absence of missplicing of the corresponding transcript, and it correlates with disease severity (101). Sustained expression of Staufen1 in the skeletal muscle of overexpressing transgenic mice causes muscle weakness and myopathy, characterized by an increase in the frequency of small fibers and central nuclei. Staufen1 impairs muscle differentiation through enhanced translation of c-myc (102), which in turn upregulates the transcription of the *PTEN* tumor suppressor gene and ultimately inhibits downstream AKT signaling (103). The AKT pathway promotes cell survival, proliferation and growth and mediates cell metabolism, transcription and translation in response to extracellular stimuli and changes in energy balance (104). The increased expression of atrogenes in Staufen1-overexpressing mice was linked to AKT signaling inactivation and PTEN upregulation, which interfere with the activity of the ubiquitin-proteasome system to promote catabolic protein degradation, which likely contributes to the muscular phenotypes (103). In further support of elevated protein degradation in DM1 muscle weakness and myopathy, DMSXL mice show enhanced proteasome activity in association with muscle weakness and myopathy (26, 105).

The dysregulation of the adaptive switch between catabolic and anabolic states in DM may extend beyond AKT missignaling, and encompass other intermediates. Maintaining an adequate supply of energy is an essential requirement for cell function, notably in muscle and CNS, which depends on the cross talk between AKT and AMPK signaling pathways (104). Interestingly, the activation of AMPK signaling is also impaired in the skeletal muscle of HSA^LR^ mice following fasting (106), corroborating the idea that DM perturbs cell master sensors of energy balance. Importantly, pharmacological treatments to normalize this pathway improved muscle strength and corrected myotonia in these mice (106). Although these data suggest a role of the AMPK cascade in DM1 muscle pathology, it was also noted that the pharmacological activation of AMPK reduced RNA foci in HSA^LR^ mice. Hence, it is possible that rather than a direct role on the etiology of muscle pathology, AMPK dysregulation perturbs the dynamics of CUG RNA, stabilizes foci and accentuates spliceopathy, thereby aggravating muscle manifestations. Conversely, the AMPK activator alone may simply destabilize RNA foci and lead to an amelioration of mouse phenotypes through a restoration of splicing.

Given the role of Staufen1 in neuronal dendrite arborization and synaptic development (107), it will be of interest to study the implication of Staufen1 in the neurological deficits of DM1. Both AKT and AMPK signaling pathways are implicated in multiple aspects of brain development and function, and their dysregulation has been associated with neurological disease (104, 108). Their role in DM may, however, be restricted to muscle, since no altered AKT/AMPK signaling activity was detected in DM1 neural stem cells (109). Nonetheless, these results must be confirmed in relevant DM mouse models of brain dysfunction.

### A role for inflammation in muscle pathology

Tumor necrosis factor superfamily member 12 (TNFSF12) was found upregulated in the skeletal muscle of DM5 and DM200 mice (Table 1), shortly after transgene induction and prior to the onset of muscle pathology (110). Genetic deletion of *Tnfsf12* or the inhibition of the downstream signaling cascade by anti-TWEAK antibodies improved the muscle strength of DM5 mice, demonstrating the physiological relevance of TWEAK signaling in DM1. The binding of TWEAK to its receptor, TNFSF12, regulates cell proliferation, differentiation, inflammation and apoptosis (111). In muscle, the TWEAK-TNFSF12 complex becomes particularly engaged in response to disease, triggering the activation of pro-inflammatory responses that can contribute to DM1 myopathy (110). Further support of ongoing inflammation in muscle was provided by global analysis of gene expression in congenital DM1, which revealed significant upregulation of pro-inflammatory genes (112).

It is conceivable that muscle weakness and atrophy in DM1 is multifactorial process, resulting not only from simultaneous dysregulation of splicing, unbalanced protein synthesis/degradation, but also inflammation.

### Changes in miRNA levels: defective transcription or maturation?

miRNA profiling revealed significant changes in the heart (113), skeletal muscle (114–118) and serum (119) of DM1 and/or DM2 patients. Despite the divergence of some of the results reported, miRNA dysregulation emerged as a disease feature, which could either be a direct consequence of RNA toxicity, or a lateral event secondary to altered cell physiology. The investigation of miR-1 dysregulation favored the former. Mature miR-1 appears to be downregulated in DM1 and DM2 hearts, in association with an expected increase in miR-1 downstream targets: the upregulation of GJA1 (connexin 43) gap junction protein and CACNA1C calcium channel might subsequently contribute to heart phenotypes (113). In an effort to shed light onto the mechanisms of miR-1 misregulation, MBNL1 knocking down in cell culture blocked the maturation of pre-miR-1, which suggested a role of MBNL1 in miRNA processing and biogenesis, in agreement with the normal or elevated levels of pre-miR-1 found in DM1 and DM2 patients, respectively (113). However, this hypothesis is at odds with subsequent findings. First, miR-1 remained unaltered in *Mbnl1* KO mice (93). It is possible that MBNL2 upregulation in these mice (21), which compensates for the lack of MBNL1, could avoid miR-1 downregulation. To answer this question it would be important to study miR-1 levels in *Mbnl1/Mbnl2* DKO. Second, global analysis of miRNA species revealed that CUG-associated changes occurred already at the precursor stage in the induced EpA960 mouse model, arguing against a primary defect in subsequent miRNA processing and maturation. Instead, these results were consistent with defects in miRNA transcription and were attributed to the dysregulation of the MEF2 transcriptional program (93). It is possible that the high expression levels of the expanded (and interrupted) transgene in EpA960 mice trigger severe molecular defects and more pronounced dysregulation of miR-1 transcription, upstream from processing and maturation, relative to DM1 and DM2 patients. Finally, recent findings on CELF1-overexpressing mice did not fully match previous results in human tissue either. In contrast with the upregulation of miR-1 targets reported in DM1 and DM2 hearts (113), GJA1 protein levels decrease in the heart of CELF1-overexpressing mice (120). The discrepancy between patients and these mice might be explained by a combined effect of the heterogeneous regional distribution of GJA1 in disease hearts, and the study of different disease stages: GJA1 levels may show an initial compensatory increase during the early adaptation disease stages studied in human samples (113), followed by a late decrease during maladaptation disease stages, like in CELF1-overexpressing mice (120). Further studies are required to clarify these questions and to extend the implications of miRNA metabolism to other affected tissues, notably the CNS.

### DM1 cardiac function: revisiting DMPK loss of function

The sequestration of expanded *DMPK* RNA in the nucleus of DM1 cells causes a 50% reduction in protein levels (121). Initial reports suggested a role of *DMPK* haploinsufficiency in disease etiology, a hypothesis corroborated by a dose-dependent effect in mouse heart: the deletion of one copy of the murine *Dmpk* gene was sufficient to disrupt cardiac conduction (122, 123). In contrast, late and mild myopathy in skeletal muscle required full deletion of both *Dmpk* copies in knockout mice (124). These early findings suggest that therapeutic hopes aiming to eliminate *DMPK* transcripts may aggravate some aspects of the disease pathology, particularly in heart. In this context, it is worth reviewing our actual knowledge on the contribution of DMPK protein to disease.

The recent re-evaluation of the impact of *Dmpk* deletion in knockout mice, bred onto homogeneous genetic backgrounds, showed no functional impact on cardiac or skeletal muscle, thereby excluding a role of DMPK loss of function in muscle phenotypes (125). The reasons behind the diverging results relative to early findings may relate to the strain background and the role of unidentified modifiers. Alternatively, the differences may relate to the replacement strategy used to inactivate the *Dmpk* gene, which might have interfered with the expression of flanking genes in the knockout lines previously generated (125). In summary, these data provide evidence of the limited functional impact of *DMPK* inactivation on heart and skeletal muscle, and validate the anti-sense therapies being developed, which are discussed below. Nonetheless, the role of DMPK protein in the CNS, as well as in other tissues, needs to be further explored.

## DM mouse models of nervous system dysfunction

The burden of CNS dysfunction has shifted DM research from an initial focus on muscle pathology, to the investigation of brain disease mechanisms. Sophisticated imaging techniques have characterized structural and metabolic abnormalities in human brains (2, 126). Molecular studies have also been performed in the nervous system, but they rely on samples collected at the end-stage of the disease. Animal models overcome this critical limitation, as they provide tissue samples throughout disease progression, offering the possibility to characterize molecular, cellular and electrophysiological changes in the nervous system prior to the onset of disease symptoms. In this section we critically review relevant neurological phenotypes of various DM mouse models, and the insight they provide to the understanding of disease mechanisms in the central and peripheral nervous system.

### The expression of toxic RNA in the CNS

Two DM1 mouse models express large CUG RNA transcripts in the CNS: the ubiquitous DMSXL line and the inducible EpA960 mice (Table 1). Both DMSXL and forebrain-induced EpA960 mice show impaired spatial learning and memory in the Morris Water Maze, resembling the visuoconstructive defects in DM1 patients (27, 31). DMSXL mice have also shown signs of anhedonia and novelty inhibition of exploratory activity (27). The electrophysiological profiling of the hippocampus revealed synaptic dysfunction behind these phenotypes: while DMSXL mice show impaired short-term paired-pulse facilitation (27), suggestive of pre-synaptic dysfunction; EpA960 exhibit reduced long-term potentiation (LTP) (31), which is more often associated with post-synaptic abnormalities (Figure 2). The diverging effects on pre- and post-synaptic neuronal plasticity between the mouse lines may be accounted for, at least partly, by their intrinsic differences: DMSXL mice express pure CUG repeats in multiple brain cell types from an early embryonic stage; while induced EpA960 mice express higher levels of interrupted CUG repeats post-natally, in the neurons of the forebrain (Table 1).

Typical RNA foci accumulation and co-localization with MBNL1 and MBNL2 were detected in various cell types of DMSXL brains (27) and in EpA960 neurons (31). Still, both lines showed only limited spliceopathy (27, 31). In contrast, *Mbnl2* KO and *Mbnl* DKO displayed more pronounced splicing dysregulation, which may contribute to impaired LTP and spatial learning of *Mbnl2* knockout mice (19, 59): the missplicing of *Grin1* may reduce dendritic localization of the glutamate receptor, which may be further aggravated by *Tanc2* abnormalities (127, 128); while *Cacna1d* and *Ndrg4* misregulation might impair neuronal activity and learning (129, 130) (Figure 2). Other MBNL-dependent pathways may, however, contribute to brain disease, such as defects in APA (59) and changes in the expression and phosphorylation of synaptic proteins (27, 50, 54).

MAPT/Tau protein has long been associated with DM1 brain disease. Abnormal MAPT isoform distribution was first described at the protein level (131), in association with the intranuclear accumulation of hyperphosphorylated protein fibers, or tangles in patients (Figure 2). Abnormal missplicing was later described in patients (46, 132) and in the brain of DMSXL mice (27). The pronounced *Mapt* RNA missplicing in *Mbnl1/Mbnl2* DKO indicates the critical role of the spliceopathy resulting from the dual loss of these two RNA-binding proteins (59). The DM1 tauopathy has been suggested to interfere with axonal transport and neurosecretion (133), but further animal studies are required to decipher the mechanisms.

## Regional distribution of DM pathology in the brain

**Figure 3:**
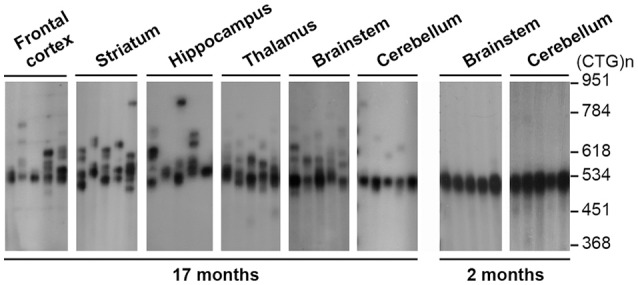
Analysis of CTG somatic mosaicism in the CNS of transgenic mice carrying the DM1 locus. The autoradiographs show representative SP-PCR analyses of 10–20 transgene molecules per reaction in dissected brain regions of old and young DMSXL hemizygotes, aged 17 and 2 months, respectively. The size markers, converted into repeat number are displayed on the right.

Imaging and neuropsychological assessment have uncovered candidate brain regions primarily affected by DM. The identification of critical brain areas will be important to direct future therapies toward the most relevant brain territories, and it will likely depend on an intricate interplay of factors, such as somatic repeat length, levels of toxic RNA, foci abundance and the activity of RNA-binding proteins. A small number of studies has investigated repeat instability (132) and *DMPK* gene expression in different brain areas in a limited number of human patients (134). DM1 mice offer the possibility to surmount the limited availability of human tissue and perform more detailed analyses. Transgenic DM1 mice expressing ~500 CTG repeats under the control of the human *DMPK* promoter and the regulatory regions of the DM1 locus (14) showed age-dependent accumulation of larger repeat sizes in most brain regions (Figure 3). The semi-quantitative results did not reveal brain regions with exceptionally high somatic mosaicism, in which we could anticipate the accumulation of very long CUG repeats. The cerebellum, however, exhibited lower levels of somatic instability, as reported in humans (135) and in another model of CTG repeat instability (136). The average repeat size in the cerebellum was nonetheless within the disease-associated range. It is possible that future analyses of somatic repeat instability in smaller brain areas or individual cell types of these mice will reveal susceptible cell populations that accumulate significantly longer repeat expansions.

Similarly, the expression levels of the *DMPK* transgene showed modest variation between CNS regions (54). In contrast, RNA foci were not homogenously distributed and accumulated preferentially in the frontal cortex and certain areas of the brainstem of DMSXL mice (26, 54), and they appeared to be more abundant in cortical astrocytes relative to neurons (27). The analysis of well-defined histological layers of the mouse cerebellum has also shown greater foci accumulation and more severe spliceopathy in Bergmann astrocytes, relative to the neighboring Purkinje cells (50). Together these findings demonstrate more pronounced pathologic events in defined brain cell populations and cell types, a view further supported by the preferential accumulation of anti-sense RAN-translated products in the oligodendrocytes of DM2 brains (70).

The factors governing the distribution of DM pathology in the brain remain elusive and must be addressed in future mouse studies, but variations in the expression of MBNL and other RNA-binding proteins between brain regions (54) and cell types should be considered (50).

## The role of GABA, glutamate and glia in DM1 neuronal hyperexcitability

While waiting for efficient gene therapy to correct the causing genetic defect (the DNA repeat expansion) or neutralize the pathogenic molecule (the toxic RNA), one can imagine pharmacological means to ameliorate or prevent progression of neurological symptoms. Such strategies require comprehensive characterization of neuronal activity and integrative brain dysfunction.

Perturbed balance between excitatory and inhibitory neurons disrupts cognition in neurological diseases. Several mouse studies favor a scenario of neuronal excitability in DM1. Both DMSXL and *Mbnl2* KO mice present elevated susceptibility to PTZ-induced seizures, suggesting GABA-mediated hyperexcitability (19). In line with elevated neuronal excitability, *Mbnl2* KO mice show augmented responsiveness to intracortical train stimulation, in a mechanism partially mediated by abnormal glutamate neurotransmission (137). Reduced expression of the glial GLT1 glutamate transporter in DMSXL brains is associated with elevated neuronal firing *in vivo* (50) (Figure 2). This finding supports a role of defective glutamatergic transmission and neuronal excitability in DM1, mediated by abnormal neuroglial interactions. Neuronal hyperexcitability is a frequent cause of epilepsy. Although epileptic episodes are rare in DM, patients present high sensitivity to GABA agonists (138) as well as abnormalities in glutamatergic transmission in the frontal lobe (139).

In addition to GABA and glutamate, circumstantial evidence points to the involvement of other signaling molecules. HPLC quantification revealed region-specific defects in dopamine and serotonin neurochemicals in DMSXL brains, in association with high foci content in dopaminergic and serotonergic brain centers (27). Importantly, DM1 brains have shown loss of neurons signaling through these two types of neurotransmitters (140, 141).

Today optogenetics allows the neuronal manipulation of neuronal circuits *in vivo*. In combination with electrophysiology, imaging and behavior assays, these techniques can provide insight into the contribution of neuronal activity to the cognitive performance of DM mice, and elucidate the neuronal circuits most profoundly affected by the disease.

### Structural, developmental and functional features of DM brains: insight from mouse models

The brain structural changes found in DM1 and DM2 are mainly characterized by white matter hyperintensities, some general atrophy and dispersed gray matter reduction across the four cortical lobes, the basal ganglia, and cerebellum. Importantly, white matter abnormalities correlate with disease duration and cognitive deficits (2, 126). Functional imaging revealed low glucose uptake and cerebral hypoperfusion, as well as abnormal connectivity patterns that correlate with atypical personality traits and executive dysfunction (142, 143). The correlation between imaging data and neuropsychological profiles hints to the involvement of complex neuronal networks, through defective neurodevelopment, neurodegeneration or neurodysfuntion. Today we still do not know the contributing weight of each of these components to DM brain disease. The molecular and histological mouse studies have shed some light on this question.

Higher expression of embryonic splicing isoforms in the brains of DMSXL (27), *Mbnl2* KO and *Mbnl1/Mbnl2* DKO mice (19, 59) points to a disrupted developmental program. In contrast, the dysregulation of synaptic proteins does not recreate embryonic events (54), supporting functional deficits in DM brains, rather than a developmental delay.

Inducible EpA960 mice have recently given further insight. Transgene induction in adult forebrain (after the completion of CNS development) yielded progressive loss of axonal and dendritic integrity, together with brain atrophy (31)—a sign of ongoing neurodegeneration in adults, possibly in line with the reported premature and accelerated cognitive decline in DM1 patients (144). However, the EpA960 mouse data do not exclude developmental disruption, should toxic RNA be expressed during early embryonic stages.

Understanding the contribution of defective development, neurodysfunction and neurodegeneration is critical to design therapeutic schemes: we must intervene prior to the establishment of irreversible developmental defects, irreparable cell damage or permanent network dysfunction. Mouse models, and in particular the inducible lines (Table 1), will help assess the reversibility of neurological phenotypes and whether neurological disease progression can be halted and even reversed.

### The peripheral nervous system and the neuromuscular junction

The involvement of the peripheral nervous system (PNS) and the presence of peripheral neuropathy in DM1 has been open to debate (145). The scarce availability of human samples has slowed down research on this topic, but mice expressing toxic CUG repeats in the PNS and in the neuromuscular junction (NMJ) have surpassed this limitation.

Axonopathy was detected in the DMSXL sciatic nerves, characterized by smaller nerve sections, loss and reduced size of myelinated fibers, in association with thinner myelin sheaths, which may highlight ongoing pathogenicity in myelinating cells. The neuronopathy extends to the spinal cord of DMSXL, where a reduction in the number of motor neurons was reported (146).

The analysis of the neuromuscular junction (NMJ) in DM1 muscle biopsies revealed abundant accumulation of RNA foci both in pre-synaptic motoneurons and in post-synaptic nuclei, with pronounced MBNL1 sequestration (147). As a result the NMJ is at risk of developing DM1-associated spliceopathy, but we currently do not know which MBNL1-dependent targets and pathways are dysregulated. In addition, the expression of two members of the SLITRK family of membrane proteins is dysregulated in DM1, in a MBNL1-independent manner, affecting neuromuscular connections (148). Together these findings suggest that both MBNL-dependent and MBNL-independent mechanisms may disturb the organization, stability and function of the NMJ, thereby contributing to PNS pathology and, importantly, to muscle pathology. In support of this view, the expression of expanded CUG RNA in the diaphragmatic NMJ of DMSXL mice is associated with disorganized endplates, lower density of postsynaptic acetylcholine receptors and reduced number of myelinated neurons, possibly mediating the respiratory impairment of these mice (28). In contrast, HSA^LR^ transgenic mice exhibit poor foci accumulation in subsynaptic nuclei (147), indicating that the muscle phenotypes of this line (such as myotonia, central nuclei and ring fibers) do not require the expression of toxic RNA in the NMJ. Subsynaptic RNA toxicity in the NMJ would preferably contribute to DM1 muscle features that are not detected in HSA^LR^ mice, such as angular fiber atrophy and pyknotic nuclear clumps (147). In conclusion, defective communication between nerve endings and skeletal muscle might be a common feature in DM1, likely contributing to muscle pathology.

## Therapy development: pre-clinical mouse studies

Following the identification of CUG repeats as the pathogenic element in DM1, expanded RNA transcripts became an attractive therapeutic target, endorsed by the reversion of disease phenotypes in an inducible mouse model of DM1 (17). Hence, the neutralization of CUG repeats has been tested in relevant DM1 mouse models, taking advantage of antisense oligonucleotides (ASO) or small molecules (Table 3).

**Table 3:** Therapeutic strategies tested in DM1 mouse models.

**Compound**	**Target**	**Administration**	**Mechanism**	**Mouse model**	**Benefits reported in DM1 mice**	**References**
**ANTISENSE OLIGONUCLEOTIDES**						
PS	CUG sequence	Intramuscular injection (local)	Steric hindrance and foci dispersion	DMSXL^a^ HSA^LR^	Dispersion of RNA foci Reduction of CUG RNA Splicing correction	(149)
Morpholino	CUG sequence	Intramuscular injection and electroporation (local)	Steric hindrance and foci dispersion	HSA^LR^ DMSXL^a^	Reduction of CUG RNA Splicing correction Mitigation of myotonia	(150)
MOE-gapmer	Flanking region	Subcutaneous injection (systemic)	RNase H-mediated degradation	HSA^LR^	Reduction of CUG RNA Splicing correction Improved histology Sustained mitigation of myotonia	(12)
MOE-gapmer	CUG sequence	Intramuscular injection and electroporation (local)	RNase H-mediated degradation	Induced EpA960	Dispersion of RNA foci Splicing correction	(151)
MOE-gapmer and morpholino	CUG sequence	Intramuscular injection and electroporation (local)	Combined RNase H and foci release	Induced EpA960	Enhanced reduction of CUG RNA	(151)
siRNA	CUG sequence	Intramuscular injection and electroporation (local)	RNAi-mediated RNA degradation	HSA^LR^	Reduction of CUG RNA Dispersion of RNA foci Splicing correction Mitigation of myotonia	(152)
cEt gapmer	*DMPK* 3′UTR	Subcutaneous injection (systemic)	RNase H-mediated degradation	DMSXL	Reduction of CUG RNA Body weight gain Improved muscle strength Improved histology	(153, 154)
siRNA	hACTA1 3′UTR	Intravenous injection of rAAV vectors (systemic)	RNAi-mediated RNA degradation	HSA^LR^	Reduction of CUG RNA Splicing correction Improved histology Mitigation of myotonia	(155)
**NUCLEIC ACID BINDING CHEMICALS**						
Pentamidine, heptamidine and diamidine analogs	CUG transcription	Intraperitoneal injection (systemic)	Inhibition of CUG transcription Foci dispersion and CUG RNA degradation	HSA^LR^	Reduction of CUG RNA Splicing correction Mitigation of myotonia	(156–158)
Hoescht derivatives	CUG-MBNL complex	Intraperitoneal injection (systemic)	Disruption of RNA foci	HSA^LR^	Splicing correction	(159)
Kanamycin derivatives	CUG-MBNL complex	Intraperitoneal injection (systemic)	Disruption of RNA foci	HSA^LR^	Splicing correction	(160)
Synthetic peptide	CUG-MBNL complex	Intramuscular injection (local)	Disruption of RNA foci	HSA^LR^	Improved histology Splicing correction	(161)
Actinomycin D	CUG transcription	Intraperitoneal injection (systemic)	Inhibition of CUG transcription	HSA^LR^	Reduction of CUG RNA Splicing correction	(162)
**PHARMACOLOGICAL APPROACHES**						
Ceftriaxone	GLT1, glial glutamate transporter	Intraperitoneal injection (systemic)	Upregulation of GLT1	DMSXL	Correction of Purkinje cell firing Improved motor coordination	(50)
Bio, Lithium, TDZD-8	GSK3ß	Intraperitoneal injection (systemic)	GSK3ß inhibition	HSA^LR^	Improved histology Improved muscle strength Mitigation of myotonia	(13)
Ro-31-8220	PKC	Intraperitoneal injection (systemic)	PKC inhibition	EpA960	CELF1 downregulation Splicing correction Amelioration of cardiac function	(88)
AICAR	AMPA signaling	Intraperitoneal injection (systemic)	AMPK activation	HSA^LR^	Dispersion of RNA foci Splicing correction Mitigation of myotonia	(106)
Rapamycin and AZD8055	mTOR signaling	Intraperitoneal injection (systemic)	mTORC1 inhibition	HSA^LR^	Improved muscle function and strength	(106)
Anti-TWEAK antibody	TWEAK/Fn14 signaling	Intraperitoneal injection (systemic)	TWEAK	DM5	Improved muscle histology Improved muscle strength Greater survival	(110)

*rAAV, recombinant adeno-associated viral; ASO, antisense oligonucleotide; BIO, 6-bromoindirubin-39-oxime; cEt, 2′,4′-constrained ethyl-modified; LNA, locked nucleic acids; MOE, 2′-O-methoxyethyl; PS, 2′-O-methyl phosphorothioate. ^a^The DMSXL mice used in these studies were hemizygous and carried ~500–800 CTG*.

### Antisense oligonucleotides to neutralize toxic RNA

ASO have been designed to disperse nuclear RNA foci and redistribute MBNL proteins, or to induce the degradation of expanded transcripts. Early approaches aimed to destabilize CUG RNA foci by direct injection of morpholino-type ASO into the skeletal muscle of HSA^LR^ mice. The reduction in nuclear foci, redistribution of MBNL1 protein and splicing correction was sufficient to improve muscle histology and myotonia (150). Similarly, 2′-O-methyl phosphorothioate (PS) modified ASO reduced foci number and corrected missplicing in two independent mouse models (149); unfortunately the molecular benefits were insufficient to improve muscle phenotypes (Table 3). Both strategies reduced the levels of toxic transcripts without RNase H activation, likely through the degradation of expanded transcripts released from nuclear foci. Alternative approaches used RNase H-active ASO to enhance nuclear RNA degradation of CUG repeats. Intramuscular injection and electroporation of 2′-O-methoxyethyl (MOE) gapmers knocked-down expanded CUG transcripts in EpA960 mice and reduced RNA foci (151). Further reduction in toxic RNA was achieved by the combination of RNase H-active MOE gampers and morpholinos (151). However, local injection caused some degree of muscle damage, which aggravated histopathology and splicing dysregulation in these mice. The systemic delivery of ASO overcomes this problem and is particularity attractive given the vast number of tissues and organs affected in DM1: systemic administration of MOE gapmers reduced expanded CUG RNA, corrected global transcriptome, ameliorated histopathology and resulted in long-term suppression of myotonia in HSA^LR^ mice (12). Similarly, 2′-4′-constrained-ethyl (cEt) ASO administrated systemically yielded robust reduction of expanded *DMPK* transcripts, improved body weight, muscle strength and histology of DMSXL mice (154). The demonstration that expanded CUG RNA is a potential target for the RNA interference (RNAi) pathway (163) suggested the therapeutic use of siRNA. Both intramuscular injection and viral delivery of siRNA molecules activated toxic CUG degradation, reduced molecular signs of RNA toxicity and improved the phenotypes of HSA^LR^ mice (152, 155).

ASO offer today a promising pipeline for therapeutic development, but their efficient delivery and biodistribution are still critical hurdles to overcome.

### Ligands and small molecules to disperse RNA foci

Small soluble chemicals with high biodistribution and low toxicity may provide an alternative to ASO. Some of these compounds were tested in DM1 mouse models (Table 3). Derivatives of pentamidine (and other diamidines), hoescht and aminoglycoside, as well as synthetic peptides yielded limited correction of missplicing in HSA^LR^ mice (156, 159, 160). While diamidines inhibit the transcription of toxic CUG RNA, the others likely disrupt RNA-protein complexes, releasing MBNL proteins from nuclear CUG foci. Although the benefits of some of these molecules were modest in mice, the results established a scaffold for chemical redesign to optimize biodistribution, reduce toxicity and increase efficacy.

Approaches limited to restoring MBNL function are unlikely to fully address the consequences of RNA toxicity and additional intermediates should also be targeted. The dissection of the molecular pathways implicated in DM1 pathogenesis revealed some of these targets and hinted at novel routes of pharmacological intervention (Table 3). In the future, therapeutic combination of multiple approaches to eliminate the primary offending RNA with approaches to correct downstream pathogenic events might be required.

### DNA as therapeutic target

Strategies targeting the DNA repeat expansion mutation were previously tested in DM1 mouse cell culture systems (164) or directly in HSA^LR^ skeletal muscle (165), and proved capable of stabilizing the trinucleotide CTG repeat tract. Although substantial effort has concentrated on the deleterious accumulation of toxic RNA, recent gene editing tools provide new means to target the upstream DNA mutation that causes DM1. CRISPR/Cas9 systems were tested in DMSXL mouse cells to induce repeat contractions (43), while modified Cas9 was used in HSA^LR^ mice to block the transcription of toxic RNA (166).

## DM1 animal models behind mice

By definition, an animal model provides a simplification of the complex human system, or at least, part of it. Mouse models offer a good compromise between easy manipulation, affordable research cost and similarity to the complex physiology of humans. However, mice have limitations too, and today there is no perfect DM1 mouse model that fully recreates all disease aspects. Conversely, reduced body mass has been repeatedly reported in mice (21, 23, 26, 77, 78) but no direct parallel has been established with human clinical symptoms, nor is it known to what extend this phenotype reflects a DM1-associated developmental delay.

Given the nature of the constructs used, transgene expression varies between models and introduces some drawbacks that should not be overlooked. Some constitutive models (such as the DMSXL mice) express low transgene levels, and require breeding to homozygosity to develop disease phenotypes. In contrast, the high expression levels in tissue-specific models (such as the EpA960 and DM5 mice) may trigger some non-specific disease features. Finally, tissue and cell type-specific expression in HSA^LR^, EpA960 and DM5/DM200 mice can mask non-cell-autonomous mechanisms, critical for some features of disease pathogenesis. In summary, the collection of mice available today covers different aspects of DM1 pathology to a certain extent, partially fulfilling the absence of a perfect mouse model, and providing means for data validation by independent laboratories.

Simple organisms can also provide complementary models for basic, translational and pre-clinical research. Although phylogenetically distant from humans, *Drosophila melanogaster, Caenorhabditis elegans* or zebrafish (*Danio rerio*), have multiple advantages over mice, including their easy manipulation, low maintenance cost and fast generation of large offspring. The expression of toxic RNA in *D. melanogaster* recreated molecular features of DM1, such as RNA foci accumulation, muscleblind protein sequestration and missplicing (167–169). Some lines showed eye degeneration (167, 168), a general readout of neurotoxicity, but which does not necessarily relate to human pathology. The development of muscle phenotypes, such as muscle wasting (167) and hypercontraction (169) seems more relevant. The expression of expanded CUG repeats also resulted in RNA foci and muscle phenotypes in zebrafish (170, 171) and *C. elegans* (172, 173). Together, these data suggest the conservation of the core mechanisms of RNA toxicity across species, and corroborate the use of simple organisms in large screenings for disease modifiers. Such studies have already resulted in the identification if genetic modifiers (167, 174, 175), chemicals that correct DM1 splicing abnormalities (176) and miRNA sponges that regulate MBNL protein levels and rescue fly phenotypes (177). The physiology of small organisms and humans are nonetheless substantially different, and therefore parallels must be established with care.

## Conclusions

Transgenic mouse models, alone or in combination, have been key to understanding fundamental molecular pathomechanisms of DM. Over the last decade, the progress in mouse studies and the advances in high throughput approaches (e.g., transcriptomics and proteomics) have led to the identification of hundreds of misregulated genes and proteins, through changes in alternative splicing, polyadenylation, protein translation and phosphorylation. Understanding the contribution of these molecular events to the etiology of DM will help depict the course between repeat expansion and the onset of disease manifestations. Future studies should continue to address “which” disease intermediates and cell populations, “where” in the tissue and “when” during disease course experience the most pronounced abnormalities. Linking these variables will identify critical events and developmental windows during which specific cell pathways are particularly sensitive to pathological insults and targetable by corrective therapies. A better understanding of pathophysiological trajectories will guide the development of efficient therapeutic approaches.

Some models have deliberately focused on specific disease features and recapitulated a small number of disease phenotypes (e.g. muscle pathology in HSA^LR^ models, cardiac function in inducible CELF1-overexpressing mice). Although oversimplifying the situation, this reductionist approach has offered the opportunity to break the complexity of disease down to tractable “building blocks” and to unravel the mechanisms behind individual aspects of the disease. The future combination of these different models, by intercrossing different transgenic lines, might be considered to “rebuild” the convoluted human disease and to explore the interdependence of individual factors. The complexity of DM pathobiology and variation in mouse models design require, however, a critical approach in the interpretation and comparison of the results obtained with different lines.

There is little doubt that mouse models will continue to provide in-depth understanding of disease. One of their major advantages is the opportunity to monitor early pathological changes, prior to the onset of disease symptoms, which is difficult to achieve in humans with the current diagnostic standards. We anticipate that future studies will uncover additional cellular pathways impacted during the disease course, while revealing targetable events to reverse disease.

## Author contributions

JA: experimental work and data acquisition; GG and MG-P: study design, interpretation, data analysis; SB and MG-P: preparation of figures; SB, GG, and MG-P: manuscript preparation.

### Conflict of interest statement

The authors declare that the research was conducted in the absence of any commercial or financial relationships that could be construed as a potential conflict of interest.
